# Total Synthesis and Anti-Viral Activities of an Extract of *Radix isatidis*

**DOI:** 10.3390/molecules191220906

**Published:** 2014-12-12

**Authors:** Li-Wei He, Hua-Qing Liu, Yu-Qing Chen, Jing-Yan Yang, Tian-Lin Wang, Wei Li

**Affiliations:** 1 School of Pharmacy, Nanjing University of Chinese Medicine, 138 Xianlin Road, Nanjing 210023, China; E-Mails: he_lw@163.com (L.-W.H.); drcoreychen@gmail.com (Y.-Q.C.); jingyan_1024@163.com (J.-Y.Y.); 2 The Second Hospital of Nanjing, 1-1 ZhongFu Road, Nanjing 210003, China; E-Mail: layuer@163.com

**Keywords:** *Radix isatidis*, isoquinoline derivative, anti-viral activity

## Abstract

*Radix isatidis* (Banlangen), a famous traditional Chinese medicine, has been used for thousands of years in China due to its anti-viral activity. Through our research, we inferred that the anti-viral activity of *Radix isatidis* depended on the water-soluble part. Among the components of this extract, the isoquinoline derivative **1** was isolated for the first time and has shown better anti-viral activity than other constituents. In this study, to solve the problem of sourcing sufficient quantities of compound **1**, a total synthesis route is described, and several analogues are also evaluated for their anti-viral activities. Among them, compound **8** shown potent anti-viral activity with an IC_50_ value of 15.3 µg/mL. The results suggested that isoquinoline derivatives possessed potent anti-viral activity and are worthy further development.

## 1. Introduction

*Radix isatidis* had a clear anti-viral effect in the clinic and has been used for thousands of years in China [1,2]. The chemical constituents are the material basis of *Radix isatidis*’s activities, but due to the complexity of its constituents, the mechanism of *Radix isatidis*’s anti-viral activity is still unknown. The main constituents include alkaloids, nucleosides, amino acids, organic acids, flavonoids, volatile oils and polysaccharides. Our group has focused on this Traditional Chinese Medicine for several years and we inferred that the anti-viral activity of *Radix isatidis* depended on the water-soluble part [3,4,5]. The isoquinoline derivative **1** was isolated from *Radix isatidis* for the first time. The anti-viral activity of compound **1** was much better than that of the other chemical constituents isolated from *Radix isatidis*, which attracted our interest [3]. Now, the most important task is to get sufficient amounts of compound **1** for the further development. In this study, a total synthesis route was design and executed. Selected compounds were also evaluated for their anti-viral activities.

## 2. Results and Discussion

### 2.1. Chemistry

**Scheme 1:**
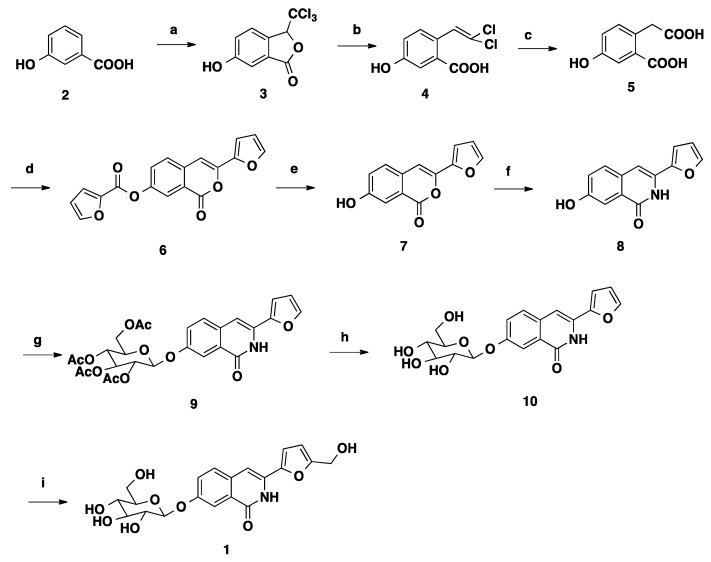
Synthesis route of 3-(5-(hydroxymethyl)furan-2-yl)-7-(((2*S*,3*R*,5*S*,6*R*)-3,4,5- trihydroxy-6-(hydroxymethyl)tetrahydro-2*H*-pyran-2-yl)oxy)isoquinolin-1(2*H*)-one (**1**).

The total synthesis route was designed according to our previous research [6] and was described in Scheme 1. 3-Hydroxybenzoic acid (**2**) was used as starting material, and was condensed with chloral hydrate in the presence of concentrated sulfuric acid to give 6-hydroxy-3-(trichloromethyl)-iso-benzofuran-1(3*H*)-one (**3**). After reduction of **3** with Zn/HOAc, 2-(2,2-dichlorovinyl)-5-hydroxy-benzoic acid (**4**) was obtained. Compound **4** was treated with concentrated sulfuric acid to give 2-(carboxymethyl)-5-hydroxybenzoic acid (**5**), followed by condensation with 2-furoyl chloride to yield 3-(furan-2-yl)-1-oxo-1*H*-isochromen-7-yl furan-2-carboxylate (**6**). Next 3-(furan-2-yl)-7-hydroxy-1*H*-isochromen-1-one (**7**) was obtained by saponification with LiOH in aqueous THF, followed by heating with ammonium hydroxide to give 3-(furan-2-yl)-7-hydroxyisoquinolin-1(2*H*)-one (**8**). 3-(Furan-2-yl)-7-(((2*S*,3*R*,5*S*,6*R*)-3,4,5-trihydroxy-6-(hydroxymethyl)tetrahydro-2*H*-pyran-2-yl)oxy)isoquinolin-1(2*H*)-one (**10**) was obtained *via* glycosidation, followed by a hydroxymethylation reaction with formaldehyde in the presence of ZnCl_2_ as catalyst to finally yield 3-(5-(hydroxy- methyl)furan-2-yl)-7-(((2*S*,3*R*,5*S*,6*R*)-3,4,5-trihydroxy-6-(hydroxymethyl)tetrahydro-2*H*-pyran-2-yl)oxy)isoquinolin-1(2*H*)-one (**1**).

### 2.2. Biological Activity

Compounds **7**, **8**, **10** and **1** were first selected to study their cytotoxicity on Vero cells by an MTT assay. Based on the results of the MTT assay, non-cytotoxic concentrations of compounds were used to determine their inhibitory effects on HSV-1 replication in Vero cells by a MTT assay. The monolayers of Vero cells in 96-well plates were infected with HSV-1 at the multiplicity of infection of 100 TCID_50_/mL. The results are shown in Table 1.

With the exception of compound **7**, all the other compounds (**8**, **10** and **1**) could, to different degrees, protect the Vero cells from HSV-1 infection. Compound **8** had most potent inhibitory effect against HSV-1, with a low IC_50_ value of 15.3 µg/mL. This result also suggested that the anti-viral activity of the aglycone derivative (compound **8**) was better than that of the glucoside derivatives (compounds **10** and **1**).

*Reagents and conditions:* (a) Chloral hydrate (1 eq), conc. H_2_SO_4,_ room temperature, 16 h; (b) Zn, HOAc, room temperature, 30 min; (c) conc. H_2_SO_4,_ room temperature, 30 min; (d) 2-furoyl chloride, 200 °C, 4 h; (e) LiOH (5 eq), THF, water, room temperature, 30 min; (f) NH_4_OH, EtOH, 120 °C, 6 h; (g) tetraacetyl-d-glucose 2,2,2-trichloroacetimidate (2 eq), CH_2_Cl_2_, BF_3_-Et_2_O, 0 °C, 2 h; (h) CH_3_ONa/CH_3_OH, room temperature, 20 min; (i) aqueous formaldehyde, AlCl_3_, 90 °C, 3 h.

**Table 1:** TC_50_ and IC_50_ values of compounds against HSV-1.

Compounds	IC_50_ (µg/mL)	TC_50_ (µg/mL)	TI *
**7**	>200	-	-
**8**	15.3	90.9	5.94
**10**	42.4	72.1	1.70
**1**	79.1	619.4	7.83
Acyclovir	1.4	-	-

* TI (Therapeutic Index) = TC_50_/IC_50_.

## 3. Experimental Section

### 3.1. Chemistry

#### 3.1.1. Materials and Solutions

All reagents were purchased from commercial sources and were used without further purification unless otherwise noted. Melting points were determined by XT-4 melting point apparatus (Beijing Tech Instrument Co., LTD, Beijing, China) and are reported without any correction. The ^1^H-NMR spectra were recorded on a Bruker AV-300 instrument (Bruker, BioSpin AG, Faellanden, Switzerland) using deuterated solvents with tetramethylsilane (TMS) as internal standard. EI-MS was recorded on a Shimadzu GCMS-2010 apparatus (Shimadzu, Tokyo, Japan). Each of the target compounds was purified by silica gel (100 mesh) column chromatography (Grace, Columbia, MD, USA). Concentration and evaporation of the solvent after reaction or extraction was carried out on a rotary evaporator (Buchi Laborterchnik AG, Flawil, Switzerland) operated at reduced pressure.

#### 3.1.2. Synthesis

*6-Hydroxy-3-(trichloromethyl)isobenzofuran-1(3H)-one* (**3**): To a solution of 3-hydroxybenzoic acid (1 g, 7.25 mmol) in concentrated sulfuric acid (3 mL) was added chloral hydrate (1.2 g, 7.25 mmol), and the mixture was stirred at room temperature for 16 h. The mixture was poured into water, and the precipitate was filtered, washed with water and dried to give compound **3** (1.4 g, 73%) as a white solid, mp 186–189 °C. MS (ESI): *m/z* calcd. for C_9_H_5_Cl_3_O_3_ [M+H]^+^ 267, found: 267. 

*2-(2,2-Dichlorovinyl)-5-hydroxybenzoic acid* (**4**): To a solution of compound **3** (0.2 g, 0.75 mmol) in HOAc (1.4 mL) was added zinc powder (0.15 g, 2.3 mmol) in portions, and the mixture was then stirred at room temperature for 30 min. The mixture was poured into water, extracted with ethyl acetate, dried over Na_2_SO_4_ and filtered to give compound **4** (0.15 g, 86%) as a white solid, mp 196–199 °C. MS (EI): *m/z* calcd. for C_9_H_6_Cl_2_O_3_ [M]^+^ 232, found: 232.

*2-(Carboxymethyl)-5-hydroxybenzoic acid* (**5**): Compound **4** (0.13 g, 0.56 mmol) was added to concentrated sulfuric acid (0.3 mL) in portions and then stirred at room temperature for 30 min. The mixture was poured into water, extracted with ethyl acetate, dried over Na_2_SO_4_ and filtered to give compound **5** (50 mg, 50%) as a yellow solid, mp 214–216 °C. MS (EI): *m/z* calcd. for C_9_H_8_O_5_ [M]^+^ 196, found: 196.

*3-(Furan-2-yl)-1-oxo-1H-isochromen-7-yl furan-2-carboxylate* (**6**): Compound **5** (6 g, 30.6 mmol) was added to furoyl chloride (17.5 mL) and the mixture was stirred at 200 °C for 4 h. The mixture was cooled down and purified via fresh column to give compound **6** (7.7 g, 78%) as a yellow solid, mp 178–180 °C; ^1^H-NMR (DMSO-*d*_6_) δ: 8.14 (s, 1H, 8-H), 8.02 (d, 1H, *J* = 2.28 Hz, 4-H), 7.93 (d, 1H, *J* = 1.08 Hz, 3'-H), 7.81 (d, 1H, *J* = 2.3 Hz, 6-H), 7.78 (d, 1H, *J* = 2.31 Hz, 5-H), 7.63 (dd, 1H, 4'-H), 7.22 (s, 1H, 5''-H), 7.03 (d, 1H, *J* = 3.39 Hz, 4''-H), 6.83 (dd, 1H, *J* = 1.71, 1.74 Hz, 3''-H), 6.72 (dd, 1H, *J* = 1.77, 1.80 Hz, 5'-H); MS (ESI): *m/z* calcd. for C_18_H_10_O_6_ [M+H]^+^ 323, found: 345 [M+Na]^+^. 

*3-(Furan-2-yl)-7-hydroxy-1H-isochromen-1-one* (**7**): To a solution of compound **6** (7.7 g, 23.9 mmol) in MeOH (105 mL), THF (310 mL) and water (105 mL) was added LiOH (5.1 g, 120 mmol), the mixture was stirred at room temperature for 30 min. The mixture was then evaporated to give compound **7** (3.7 g, 68%) as a yellow solid, mp 173–175 °C; ^1^H-NMR (DMSO-*d*_6_) δ: 10.54 (s, 1H, 7-OH), 7.86 (s, 1H, 8-H), 7.48 (d, 1H, *J* = 2.4 Hz, 6-H), 7.38 (dd, 1H, *J* = 4.4, 3.3 Hz, 4'-H), 7.29 (dd, 1H, *J* = 1.77, 1.8 Hz, 3'-H), 7.07 (s, 1H, 4-H), 6.9 (d, 1H, *J* = 3.36 Hz, 5-H), 6.67 (dd, 1H, *J* = 1.77, 1.8 Hz, 5'-H); MS (ESI): *m/z* calcd. for C_13_H_8_O_4_ [M+H]^+^ 229, found: 251 [M+Na]^+^.

*3-(Furan-2-yl)-7-hydroxyisoquinolin-1(2H)-one* (**8**): To a solution of compound **7** (3.7 g, 16.2 mmol) in ethanol (15 mL) was added NH_4_OH (15 mL), the mixture was stirred at 120 °C for 6 h. The mixture was then cooled down and evaporated. The residue was added small amount of water, extracted with ethyl acetate, dried over Na_2_SO_4_ and filtered to give compound **8** (2.9 g, 79%) as a yellow solid, mp 135–137 °C; ^1^H-NMR (DMSO-*d*_6_) δ: 11.33 (s, 1H, 2-H), 9.99 (s, 1H, 7-OH), 7.8 (d, 1H, *J* = 1.2 Hz, 8-H), 7.6 (d, 1H, *J* = 8.58 Hz, 5-H), 7.52 (d, 1H, *J* = 2.52 Hz, 6-H), 7.27 (d, 1H, *J* = 3.42 Hz, 4'-H), 7.2 (dd, 1H, *J* = 2.61, 2.64 Hz, 5'-H), 6.88 (s, 1H, 4-H), 6.63 (dd, 1H, *J* = 1.77 Hz, 1.8, 3'-H); MS (ESI): *m/z* calcd. for C_13_H_9_NO_3_ [M+H]^+^ 228, found: 250 [M+Na]^+^.

*3-(Furan-2-yl)-7-(((2S,3R,5S,6R)-3,4,5-trihydroxy-6-(hydroxymethyl)tetrahydro-2H-pyran-2-yl)oxy)isoquinolin-1(2H)-one* (**10**): To a solution of compound **8** (1.5 g, 6.6 mmol) in CH_2_Cl_2_ (100 mL) was added tetraacetyl-d-glucose 2,2,2-trichloroacetimidate (6.5 g, 13.2 mmol), the mixture was then added BF_3_-Et_2_O at 0 °C and stirred for 2 h. The mixture was added Et_3_N, evaporated and purified via fresh column to give a yellow solid. The solid was then added CH_3_ONa/CH_3_OH (2 mL) and the mixture was stirred at room temperature for 20 min. The precipitate was then filtered, washed with methanol, dried to give compound **10** (1.65 g, 92%) as a white solid, mp 223–226 °C; ^1^H-NMR (D_2_O) δ: 7.82 (d, 1H, *J* = 1.6 Hz, 8-H), 7.75 (d, 1H, *J* = 2.8 Hz, 5-H), 7.7 (d, 1H, *J* = 8.8 Hz, 6-H), 7.42 (dd, 1H, 4''-H), 7.31 (d, 1H, *J* = 3.2 Hz, 3''-H), 6.95 (s, 1H Hz, 4-H), 6.65 (dd, 1H, *J* = 1.6, 2.0 Hz, 5'-H), 5.0 (d, 1H, *J* = 5.4 Hz, 1''-H), 3.7 (m, 1H, 6''-H), 3.52 (m, 1H, 6''-H), 3.41 (m, 1H, 2''-H), 3.33 (m, 1H, 3''-H), 3.28 (m, 1H, 5''-H), 3.2 (t, 1H, 4''-H); MS (ESI): *m/z* calcd. for C_19_H1_9_NO_8_ [M+H]^+^ 390, found: 390 [M+H]^+^.

*3-(5-(Hydroxymethyl)furan-2-yl)-7-(((2S,3R,5S,6R)-3,4,5-trihydroxy-6-(hydroxymethyl)tetrahydro-2H-pyran-2-yl)oxy)isoquinolin-1(2H)-one* (**1**): To a solution of compound **10** (0.1 g, 0.26 mmol) in HCHO (5 mL) was added AlCl_3_ (56 mg, 0.42 mmol), the mixture was stirred at 90 °C for 3 h. The mixture was then evaporated and the residue was purified via fresh column to give compound **1** (34 mg, 31%) as a red solid, mp 232–233 °C; ^1^H-NMR (DMSO-*d*_6_) δ: 10.43 (1H, s, H-2), 7.25 (1H, s, H-4), 6.73 (1H, d, *J* = 8.5 Hz, H-5), 6.98 (1H, dd, *J* = 8.4, 2.1 Hz, H-6), 8.06 (1H, d, *J* = 2.1 Hz, H-8), 7.22 (1H, d, *J* = 3.2 Hz, H-3'), 6.60 (1H, d, *J* = 3.2 Hz, H-4'), 4.63 (2H, d, *J* = 5.6 Hz, H-6'), 5.52 (1H, t, *J* = 5.8 Hz, 6'-OH), 4.86 (1H, d, *J* = 7.7 Hz, H-1''), 3.21 (1H, m, H-2''), 3.28 (1H, m, H-3''), 3.15 (1H, m, H-4''), 3.27 (1H, m, H-5''), 3.63 (1H, m, H-6''), 3.45 (1H, m, H-6''); MS (ESI): m/z calcd. for C_19_H1_9_NO_8_ [M+H]^+^ 390, found: 390 [M+H]^+^.

### 3.2. Anti-Virus Biological Assay

#### 3.2.1. Materials and Solutions

96-Well plates (Corning Corporation, Corning, NY, USA); Power Wave 340 type Microplate reader (Bio-Teck Corporation, Winooski, VT, USA); BC- J80S type CO_2_ Incubators (BoXun Industrial Co., Ltd., Shanghai, China); 79-1 type Magnetic heating Stirrer (Jintan Medical Instrument Factory, Jintan, Jiangsu, China); 20–200 µL and 100–1000 µL Pipette (Genex Beta, Suzhou, Jiangsu, China); Centrifuge tube (Shanghai Shen’an Medical Devices Factory, Shanghai, China); SYQ-DSX-280B type Portable Stainless Steel Pressure Steam Sterilizer (Shanghai Shen’an Medical Devices Factory, Shanghai, China); Laboratory-grade ultra-pure water Instrument (EPED, Nanjing, Jiangsu, China); LDZ5-2 type Low-speed automatic balance centrifuge (Beijing Medical Centrifuge factory, Beijing, China); KQ-500DE type CNC ultrasonic cleaner (Kunshan Ultrasonic Instrument Co., Ltd., Kunshan, China); Human herpes simplex virus type I (HSV-1) passage twice to enhance virulence (provided by Nanjing Medical University Virus Lab, Nanjing, Chana); Vero cells (sensitive cells to HSV) were routinely grown in RPMI1640 medium with 10% newborn calf serum (NCS) and 0.06 mg/mL penicillin, 0.1 mg/mL streptomycin, 2 mg/mL NaHCO_3_. Grown as a monolayer culture; 10% newborn calf serum (Lanzhou MinHai Biological Engineering Co., Ltd., a Sino-US joint venture, Lanzhou, China); culture medium RPMI (Gibco Company, Grand Island, NE, USA); Acyclovir powder (a gift of Jiangsu Provincial Hospital, Nanjing, China); 100TCID50 virus solution (prepared by Department of Microbiology and Immunology, Nanjing University of Traditional Chinese Medicine, Nanjing, China).

#### 3.2.2. Cytotoxic Effects of Compounds on Vero Cells

Test compounds were accurately weighed, fully dissolved in fresh RPMI1640 medium, filtered for sterilization, and saved at 4 °C. Vero cells were digested by trypsin, 1640 medium containing 10% NCS was added, and a 2 × 10^5^ /mL cell suspension was prepared. Confluent cell monolayers in 96-well multidishes (0.1 mL per well) were prepared at 37 °C, 5% CO_2_, and the supernatants were removed. The sample solutions were diluted to seven concentrations: 2000, 1000, 500, 100, 20, 4 and 0.8 µg/mL. The above liquid was added to 96-well plates (0.2 mL per well, each concentration with four wells), two control groups of Vero cells were also set up. MTT (0.02 mL per well) was added after culturing for 72 h at 37 °C, 5% CO_2_, then the plates were placed in a 37 °C 5% CO_2_ incubator and incubated for 4 h. The culture medium was removed, DMSO (0.2 mL per well) was added, and shaken for 5–10 min. The absorbance value was measured at the wavelength of 570–630 nm, cell survival and half toxic concentration (TC_50_) were calculated by the Reed-Muench method.

#### 3.2.3. Anti-HSV-1 Activity Assay

Maximum non-toxic concentrations were chosen as the initial concentration, and then they were diluted to several concentrations for the anti-viral experiments. Four groups were set up: normal cells control, viral infection control, test drug control, positive drug control. Acyclovir was chosen as positive drug. Monolayers of Vero cells prepared in microtiter plates were infected with 100 TCID_50_ HSV-1 diluted in PBS, 20 µL per well, while PBS 0.02 mL per well was added in the normal cell controls. After 1 h at 37 °C 5% CO_2_, residual inoculum was removed and cell monolayers were washed twice with PBS. Test compounds which had been diluted in assay medium were added to give 0.2 mL per well, and each concentration has four wells. Plates were incubated at 37 °C 5% CO_2_ for 72 h. Then MTT (0.02 mL per well) was added after culturing for 72 h at 37 °C 5% CO_2_, then the plates were placed in a 37 °C 5% CO_2_ incubator for 4 h, the culture medium was removed, DMSO (0.2 mL per well) was added and shaken for 5–10 min, and the absorbance value was measured at a wavelength of 570–630 nm. The rate of viral suppression and IC_50_ were calculated by the Reed-Muench method. The therapeutic index (TI) is calculated according to the formula TI = TC_50_/IC_50_.

## 4. Conclusions

In conclusion, this study presented a convenient total synthesis route of a new constituent (compound **1**) from *Radix isatidis*, which solves the problem of the availability of this compound. Also, some isoquinoline derivatives were tested for their inhibitory effects against HSV-1. The results show that compound **8** had potent anti-viral activity with an IC_50_ value of 15.3 µg/mL, which suggested that isoquinoline derivatives might possess potent anti-viral activity and are worthy of further development.

## Supplementary Materials

Supplementary materials can be accessed at: http://www.mdpi.com/1420-3049/19/12/20906/s1.
