# Supplementary material for: Total Synthesis and Anti-Viral Activities of an Extract of Radix isatidis
Source: Molecules. 2014 Dec 12;19(12):20906–12. doi: 10.3390/molecules191220906 (PMC6271997; doi:10.3390/molecules191220906)

Supplementary Materials

Figure S1. MS of compound **6**.

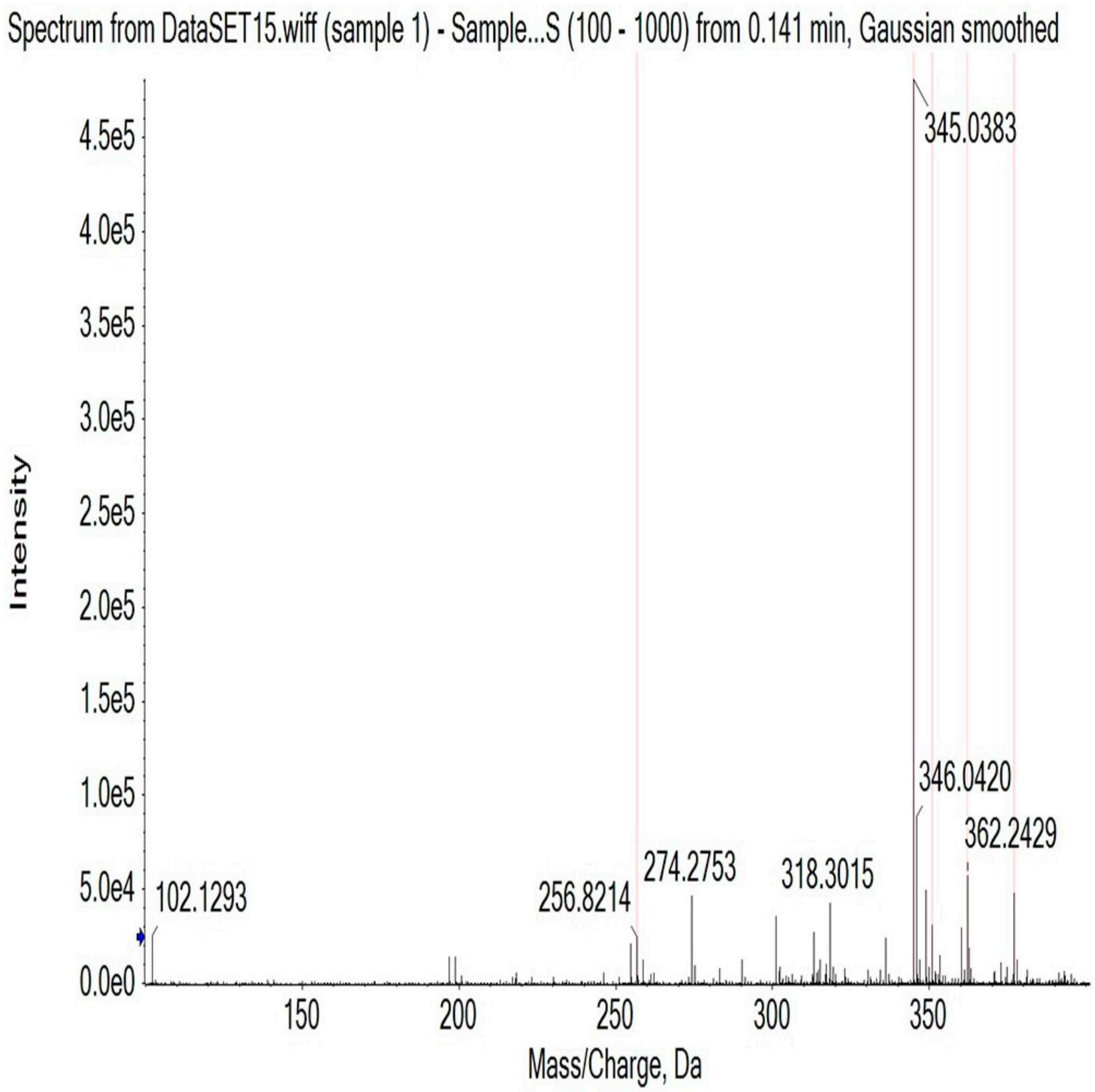

Figure S2. NMR of compound **6**.

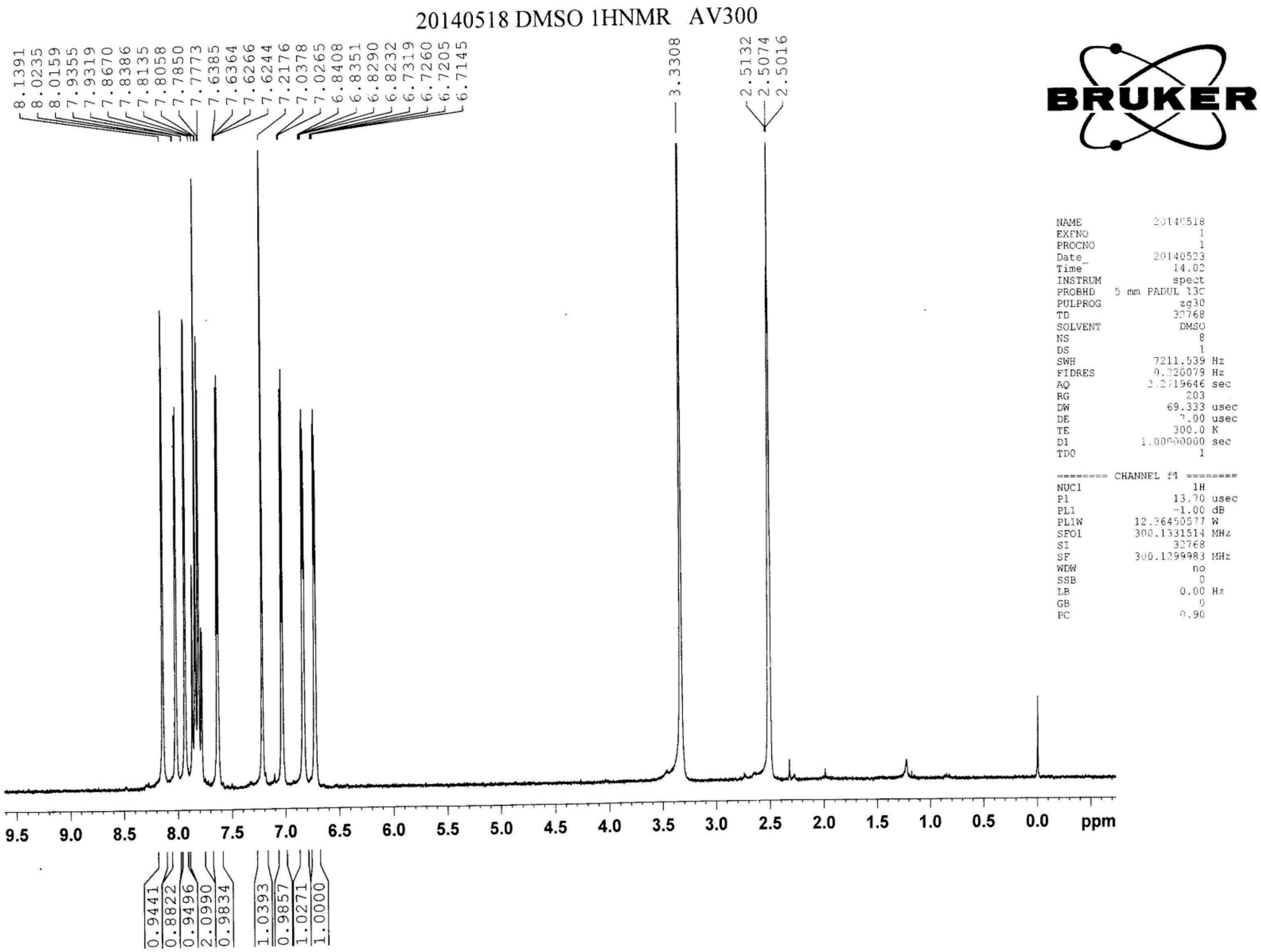

Figure S3. MS of compound **7**.

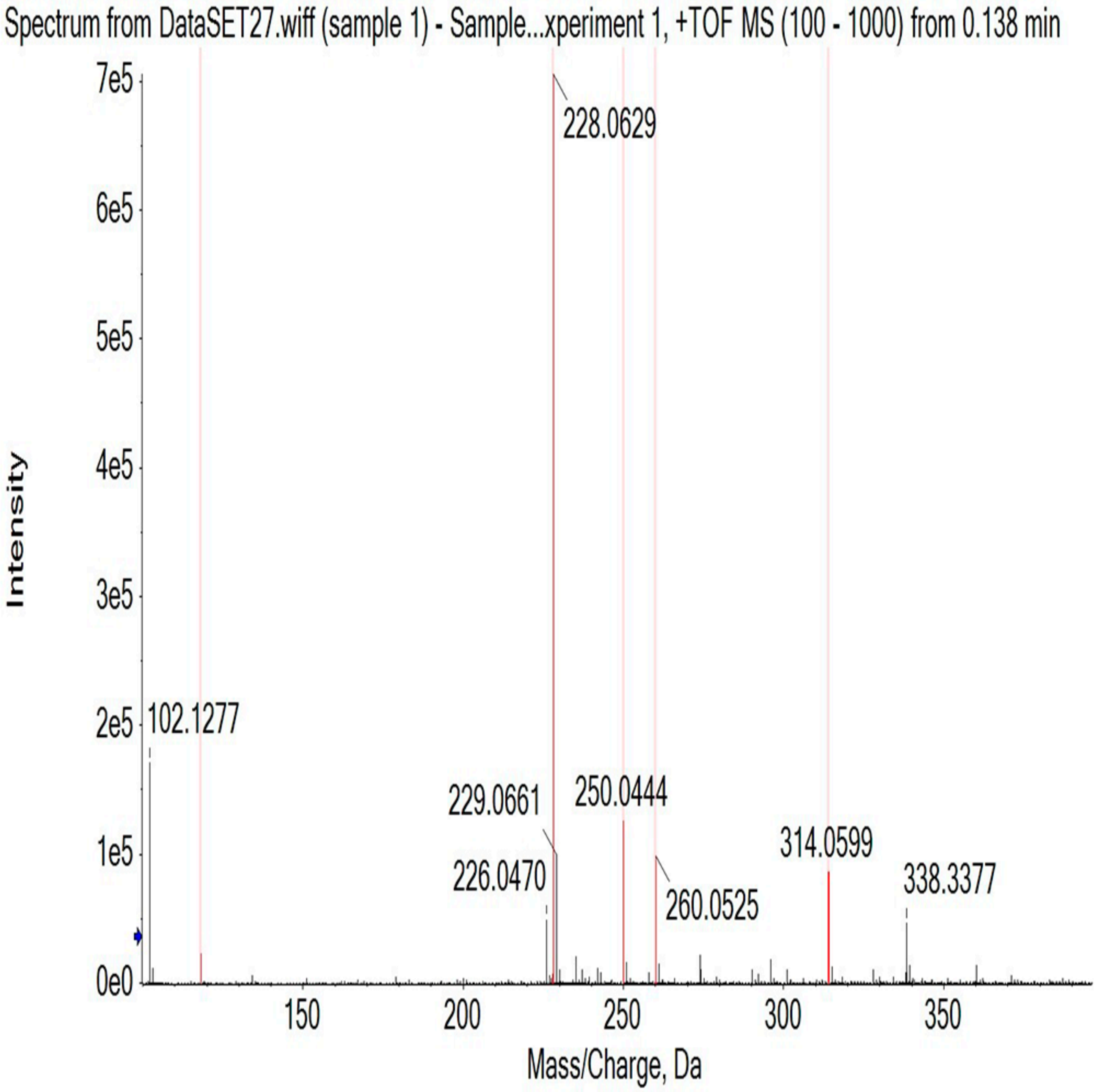

Figure S4. NMR of compound **7**.

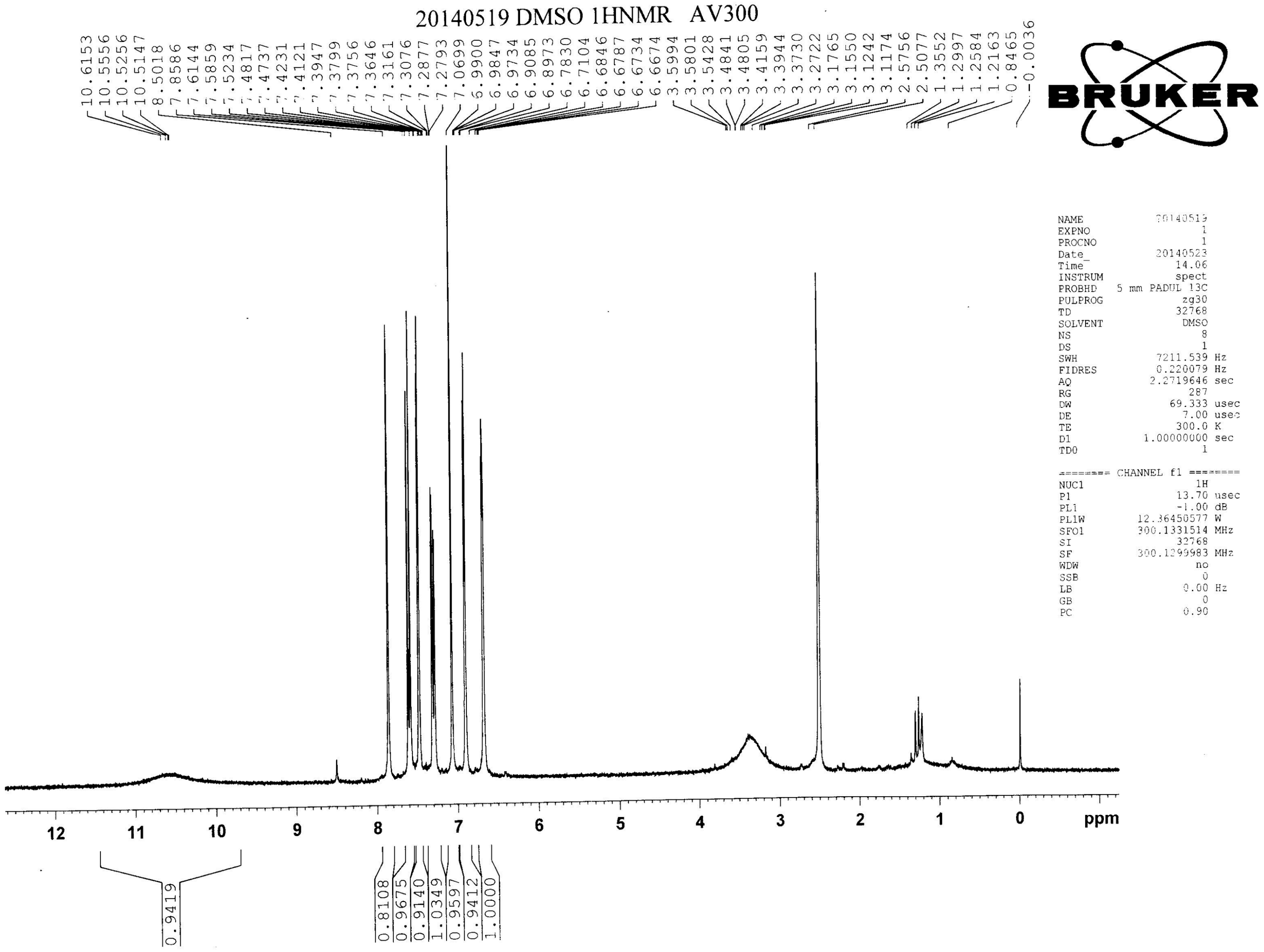

Figure S5. MS of compound **8**.

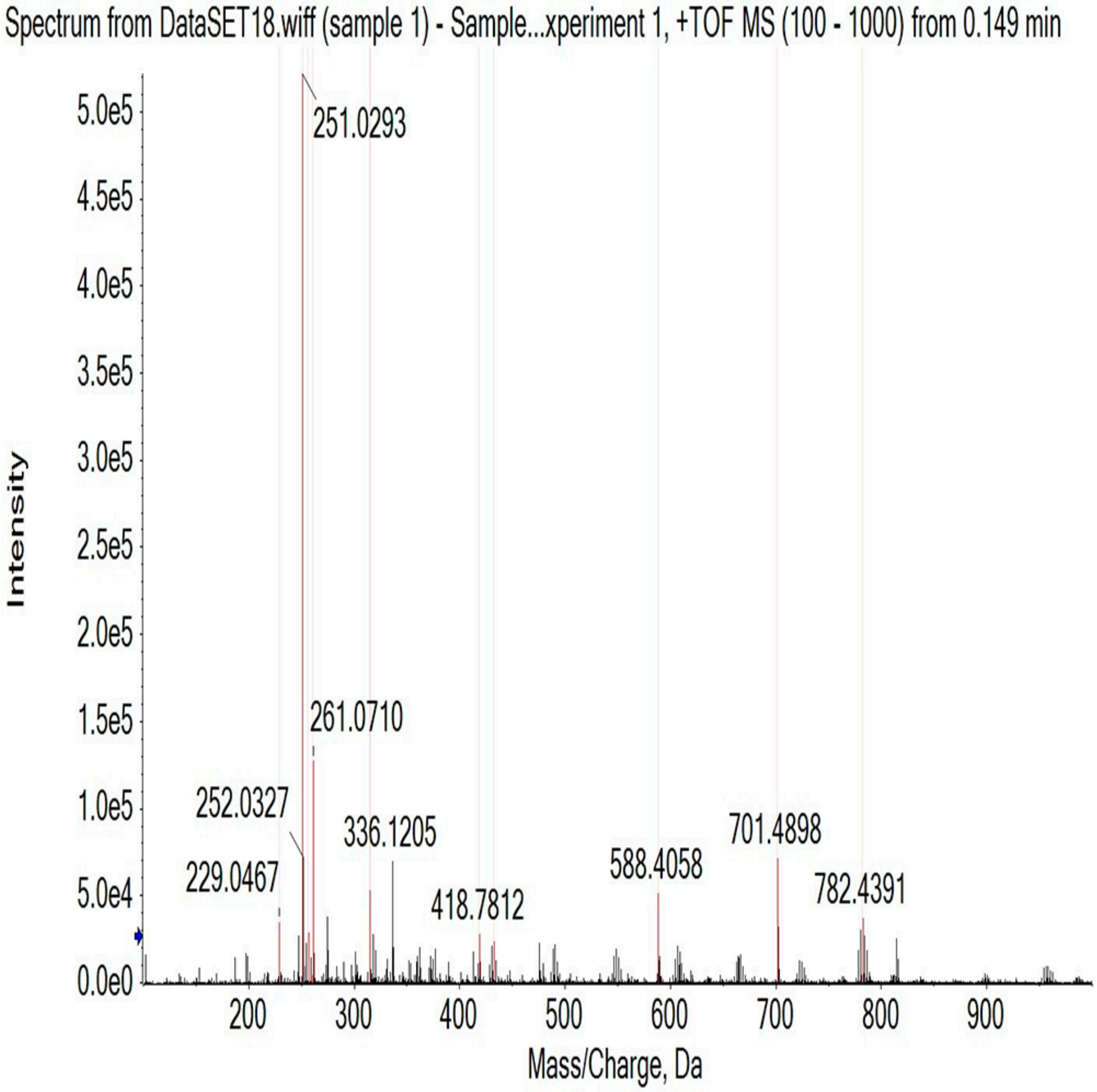

Figure S6. NMR of compound **8**.

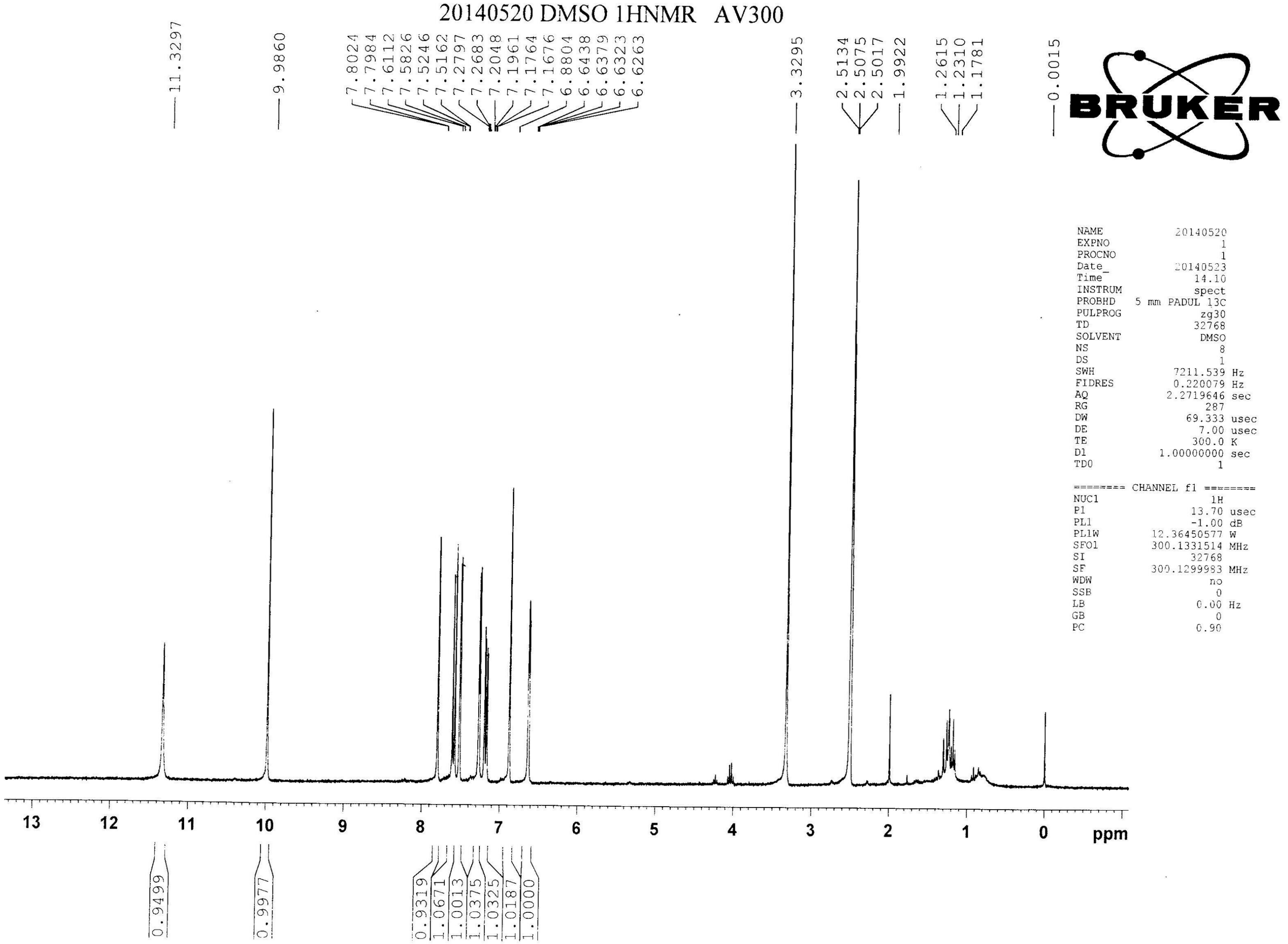

Supplement: Supplementary file 1 [file molecules-19-20906-s001.pdf]
